# Genome report: genome sequence of the hibiscus mealybug, *Nipaecoccus viridis* (Newstead), an invasive pest of citrus

**DOI:** 10.1093/g3journal/jkaf154

**Published:** 2025-07-07

**Authors:** Tracy Liesenfelt, Amanda Markee, Emilie P Demard, Lauren M Diepenbrock, Andrew J Mongue

**Affiliations:** Department of Entomology and Nematology, Institute of Food and Agricultural Sciences, University of Florida, Gainesville, FL 32611, United States; Richard Gilder Graduate School, Department of Invertebrate Zoology, American Museum of Natural History, New York, NY 10024, United States; Department of Entomology and Nematology, Institute of Food and Agricultural Sciences, University of Florida, Citrus Research and Education Center, Lake Alfred, FL 33850, United States; Department of Entomology and Nematology, Institute of Food and Agricultural Sciences, University of Florida, Citrus Research and Education Center, Lake Alfred, FL 33850, United States; Department of Entomology and Nematology, Institute of Food and Agricultural Sciences, University of Florida, Gainesville, FL 32611, United States

**Keywords:** scale insect, invasive pest, endosymbionts, Hemiptera, paternal genome elimination, genome assembly

## Abstract

Mealybugs are frequently known for being pest insects to both ornamental and large-scale agricultural crops. Yet despite their agricultural importance, the genomic resources for this group remain quite limited. One such species is the hibiscus mealybug, *Nipaecoccus viridis* (Newstead) (Hemiptera: Coccomorpha: Pseudococcidae). This invasive mealybug species has recently expanded throughout Florida and has spread across the state. Genomic resources would provide a new means to better understand the invasive nature of this insect, and thus, we present the de novo genome assembly for *N. viridis*. Our genome assembly is 289 Mb, in which 91.2% of this sequence assembled into 5 chromosomal scaffolds. We report 15,370 genes to be present within our genome. We found that repetitive elements in the genome accounted for 32.40% of the sequence. These statistics follow similar trends to other previously sequenced Pseudococcidae species.

## Introduction

Despite technology such as PacBio long-read sequencing becoming more readily available and allowing genome sequencing to become more obtainable, the sequencing for agricultural pests lags behind other sequenced insects (Li *et al.* 2019). While often small and unnoticed, mealybugs (Hemiptera: Coccomorpha: Pseudococcidae) can pose large threats to agricultural systems and inflict economically important damage to crops. This generalist crop pest lifestyle, paired with many species' highly invasive nature, make them an outsized threat to agriculture (Liebhold *et al.* 2024). The assembly of genomes for species in the family Pseudococcidae holds the key to understanding these insects at multiple levels, including their invasive pathways, endosymbiotic bacteria, and unique sex determination system.

Mealybugs can infest a large host range as the result of utilizing endosymbiotic bacteria. Like most other Hemiptera, they are plant-sucking insects that feed on plant phloem with their stylets. However, this feeding lifestyle alone is not enough to provide mealybugs with all of its necessary nutrients, such as essential amino acids; thus they utilize endosymbiotic bacteria to aid in this synthesis (Husnik *et al.* 2013; Garber *et al.* 2024). These bacteria are housed inside a specialized organ known as a bacteriome, which is a nested symbiotic arrangement in which one bacterium lives inside another together inside the cells of the insect (Husnik and McCutcheon 2016). In mealybugs, the primary Proteobacteria endosymbionts (P-endosymbionts) are various species from the genus *Tremblaya*. Previous phylogenetic analyses suggest an origin from an infection of a mealybug ancestor with a free-living *Tremblaya* precursor, ultimately leading to the cospeciation of the mealybug and endosymbiont (Baumann and Baumann 2005). Additionally, other morphologically diverse bacteria known as S-endosymbionts can be present, which can include species from the genera *Sodalis*, *Wolbachia*, *Rickettsia*, and several others (Baumann 2005). While some species of bacteria have been characterized in mealybugs, the availability of additional genomes in Pseudococcidae can aid in the understanding of how these symbiotic relationships have evolved. Beyond their unique symbiotic ecology and importance in applied agricultural research, this group of insects is also important to study from the basic research perspective.

Scale insects, which include mealybugs, exhibit many unique modes of sex determination, including the poorly understood system of paternal genome elimination (PGE), a rare form of haplodiploidy (Nur 1966; de la Filia *et al.* 2015). Under PGE, while males will inherit their genetic material from both parents, they are only able to pass on their mother's genes to their offspring (Herbette and Ross 2023). Within mealybugs, one set of the genome is deactivated. This occurs through heterochromatinization during their early development, and while this inactive set is maintained in somatic cell lines, it is not passed onto their offspring as it is destroyed in meiosis before it can end up in mature sperm (Nur 1990; Mongue *et al.* 2025).

**Fig. 1.**
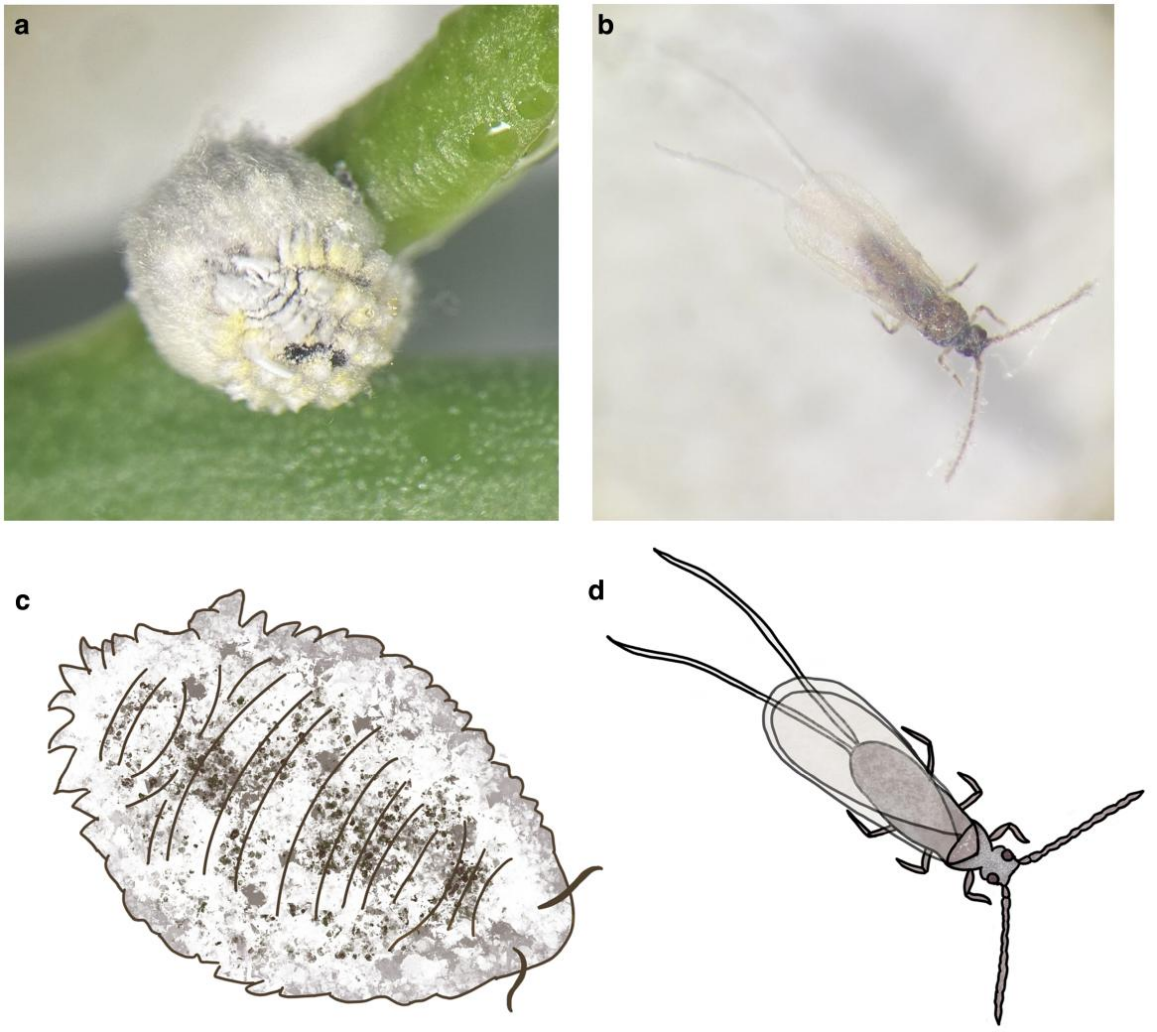
Depictions of sexual dimorphism present in adult *N. viridis*. a) An adult female covered in wax with her ovisac underneath her body. b) A winged adult male. c and d) Illustrations depicting the adults of *N. viridis* to highlight their sex differences. Photos by TL, illustrations by TL.

In service of future study of mealybugs, we present the genome of an agricultural pest that recently arrived in Florida, the hibiscus mealybug *Nipaecoccus viridis* (Newstead). Adults of this species are highly sexually dimorphic (Fig. 1) having winged males with a body length of around 1.3 to 2.5 mm and adult females have soft oval-shaped body about 2.5 to 3 mm and 1.5 to 2.5 mm wide (Sharaf and Meyerdirk 1987). Adult females use waxy secretions to create an ovisac beneath their bodies to harbor their eggs; a single female produces anywhere between 400 and 1,000 eggs in its lifetime (Olabiyi *et al.* 2023a).

While their ancestral geographic range is not known, *N. viridis* is believed to have originated in Asia (Evans and Dooley 2013) and has since been spread to at least 60 different countries (García Morales *et al.* 2016). Part of this worldwide invasive success comes from their massive host range. *N. viridis* is highly polyphagous, feeding on at least 140 recorded genera of plants (García Morales *et al.* 2016). This insect can be a major pest on several economically important crops including soybeans, pomegranate, cotton, cassava, and citrus—the latter being important to the recent invasion of *N. viridis* into Florida.

**Fig. 2.**
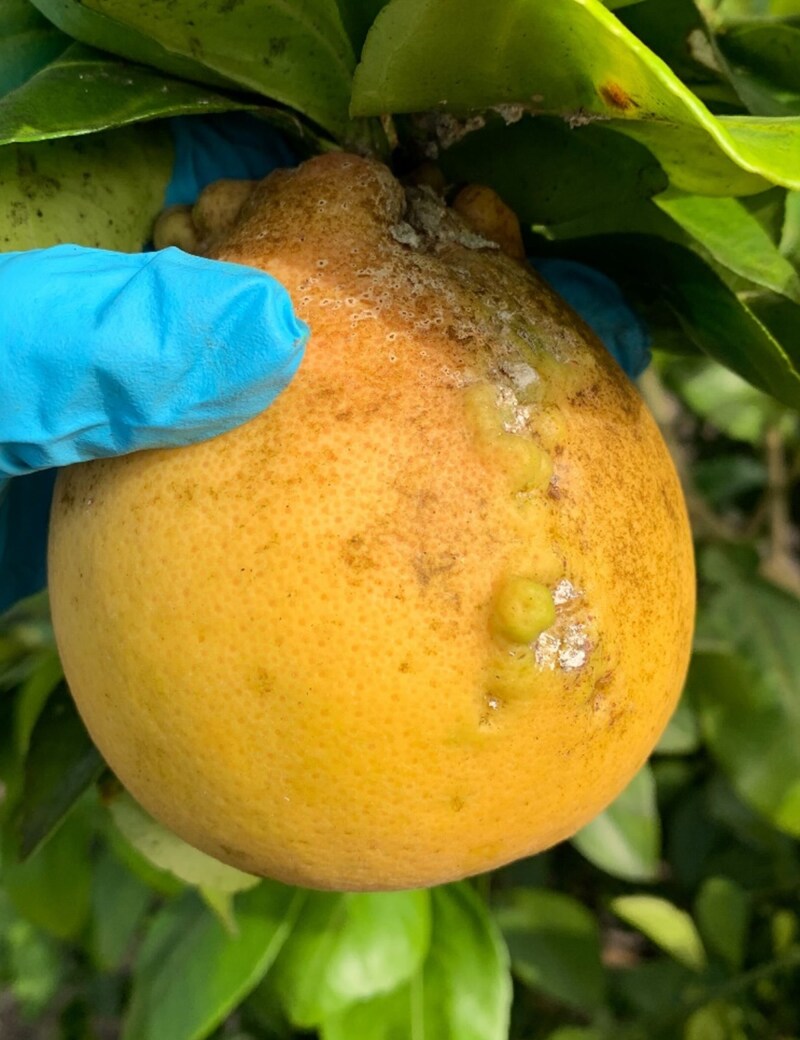
Pictures of deformations present on a grapefruit due to feeding damage from *N. viridis.* Their feeding causes gall-like outgrowths on the surface of the fruit. Along with the outgrowths, residual white wax can be seen left behind on the fruit. Photo by TL.

In 2009, *N. viridis* was first recorded on dodder (*Cuscuta exaltata*) by the Cooperative Agricultural Pest Survey in the Rosemary Scrub Natural Area of Palm Beach County, Florida (Stocks and Hodges 2010). It was not until 2019 when the mealybug was recorded in commercial citrus plots that concerns were raised regarding the damage it could pose to Florida's agriculture (Diepenbrock and Ahmed 2020). These insects pose a threat to the marketability of fresh fruit. In particular, *N. viridis* can cause a range of damage when feeding on citrus, including fruit drops, deformed fruit (Fig. 2) and even death to young trees (Diepenbrock and Ahmed 2020; Olabiyi *et al.* 2023a). These threats make tracking and mitigating its invasion an urgent priority.

The origins of this invasion into Florida are unknown, but since the first detection in commercial citrus in 2019, *N. viridis* has become widespread across the southern region of the state recorded in 24 different counties (Deeter and Ahmed 2023; Olabiyi *et al.* 2023a; personal communication with Florida Department of Agriculture and Consumer Services). Observation data alone cannot resolve invasion history, but genomic tools like a high-quality reference genome will allow researchers to explore the population history through variation in genetic markers. To facilitate this ultimate goal, as well as provide more resources on endosymbiotic bacteria characterization and a rare sex determination system, this study presents the first chromosome-level assembly of *N. viridis*.

## Materials and methods

### Collection of *N. viridis*

DNA was sourced from adult female *N. viridis* collected from a lab-reared colony. This colony was initially established in 2019 from a field-collected population taken from Highlands County, Florida (latitude: 27°20′24.00″ N; longitude: −81°20′24.00″ W) and reared in an indoor insectary at the University of Florida Citrus Research and Education Center (CREC) in Lake Alfred, Florida (25 ± 5 °C, relative humidity of 70% ± 10%, and a photoperiod of 16:8 h [L:D]; Olabiyi *et al.* 2023b). The colony has been maintained without additional introductions from the wild population and has since become a distinct, inbred line. For our genome project, adult females were collected in August 2023 from this colony, roughly 45 generations after the initial establishment based on the approximately 32-day life cycle. Under the mealybug sex determination system of PGE, both males and females share the same set of chromosomes across the genome, *i.e.* there are no sex chromosomes (Nur 1990). Therefore, the same genetic information is contained in males and females. We chose females for the convenience of larger body size than males with no trade-off in chromosomal sampling. Additionally, with the utilization of an inbred lab colony, we could pool DNA from different individuals without introducing much genetic variation. Whole live females were flash frozen in liquid nitrogen and then stored in a −80  °C freezer prior to extraction.

### DNA extraction and sequencing

#### Homogenization and DNA extraction

To obtain DNA for sequencing, we homogenized individual females using a PowerMasher II tissue disruptor (Funakoshi). This tool utilizes a motorized pestle to trap and homogenize tissue directly into a genomic lysis buffer. The DNA was then extracted using an Omniprep DNA extraction kit for high-quality genomic DNA (G-Biosciences). We used the standard Omniprep base kit protocol, with minor adjustments outlined in Mongue *et al.* (2024a), which successfully generated a chromosome-level assembly of another scale insect species. These modifications included lysing tissue overnight for approximately 15 to 16 h on an incubator (Fisherbrand, mini heat block) at 56 °C. During the DNA precipitation step, 2 μL of 20 mg/mL Mussel Glycogen (Thermo Scientific) were added to each sample to increase DNA yield. Once this reagent was added to each sample, we allowed DNA to precipitate for 1 h in a −20 °C freezer. These modifications allowed us to maximize our DNA yields and decrease contamination of samples due to the wax coating on the individual females being extracted.

#### Quality control and sequencing

We checked the quality of DNA and length fragmentation using a combination of a Qubit 4 Fluorometer with Qubit dsDNA 1X broad range Sensitivity Assay Kit and a Nanodrop spectrophotometer.

To meet sequencing core specifications, we needed a minimum total of 2 μg of DNA. To reach this threshold with such a small insect, we selected samples with at least 200 ng of total DNA based on Qubit concentration and a purity of >1.7 for 260/280 based on Nanodrop reading. In total, we submitted a pooled extraction of 9 adult females from the inbred colony to be sequenced at the University of Florida's Interdisciplinary Center for Biotechnology Research (ICBR) on a PacBio Sequel IIe platform (Menlo Park, CA, USA) for HiFi long-read sequencing. We separately pooled 500 mg of whole-body tissue from adult females, flash froze in liquid nitrogen, and submitted to Novogene Inc. (Sacramento, CA, USA) to generate Illumina Hi-C linked reads with the Illumina NovaSeq sequencing platform. In both cases, pooling across multiple individuals was necessary to meet quantity thresholds for library preparation.

### Genome size estimates

After pooling for sequencing, we did not have enough tissue for flow cytometry methods to estimate genome size. Instead, we used Jellyfish v. 2.2.4 (Marçais and Kingsford 2011), a tool that allowed us to estimate our expected genome size using the raw reads from PacBio by generating k-mer frequencies; specifically we used -m 21 to count sub-sequences of 21 bases in length. Data was analyzed using custom scripts in R Studio v. 4.2.0 (R Core Team 2018). Genome size was estimated using the following equation: Genome Size = the abundance of each k-mer (x-axis) × frequency (y-axis)/mean coverage (peak of the histogram).

### Genome assembly

#### Contig assembly from raw reads

Our primary genome was assembled on the University of Florida HiPerGator high-performance computing cluster using the program Hifiasm v.0.18 (Cheng *et al.* 2021) with the -l 3 parameter to aggressively remove haplotigs.

#### Decontamination of primary assembly

Post-assembly, we used BlobTools v.1.0 (Laetsch and Blaxter 2017) to search for potential contaminants and endosymbionts. With the endosymbionts present in the bacteriome (Husnik *et al.* 2013; Garber *et al.* 2024), we anticipated the presence of bacterial sequences in this assembly. Contigs identified by BlobTools as endosymbiont candidates were compared against the NCBI BLAST database to provide more specific identification of bacterial species but were not removed at this stage of the assembly. We also assessed the assembly for genome completeness using the hemipteran ortholog dataset (hemiptera_odb10) for BUSCO v. 5.7.0 (Manni *et al.* 2021).

#### Hi-C scaffolding to resolve chromosomes

To achieve a chromosome-level assembly, we used the results of our Hi-C sequencing in combination with the PacBio primary assembly. We aligned the Hi-C reads to the primary assembly using the Arima Hi-C pipeline (https://github.com/ArimaGenomics/mapping_pipeline) and used Picard v.2.18.2 (https://broadinstitute.github.io/picard/) to check for and remove duplicate sequences. The assembly was then scaffolded using YaHS v.1.1 (Zhou *et al.* 2023). We generated an initial scaffolded assembly and created a Hi-C contact map through the generation of a JBAT file for visualization in Juicebox v.2.3.0 (Robinson *et al.* 2018). This assembly was manually curated and corrected for misalignments and updated with YaHS “juicer post” to produce a final assembly fasta (Zhou *et al.* 2023). We again used BlobTools to identify bacterial sequences and the hemipteran ortholog dataset (hemiptera_odb10) for BUSCO v. 5.7.0 to assess the completeness of our curated assembly.

#### Identification of Endosymbionts

To identify other sequenced organisms, especially endosymbiotic bacteria within the genome, we evaluated several pieces of evidence during assembly curation. First, we analyzed the primary assembly using BLAST+ v.2.9 with the tool Megablast (Chen *et al.* 2015) to quickly compare the assembly against known sequences in the NCBI BLAST database. We took the best hit with an e-value <0.00001 for each contig. Next, we aligned the raw PacBio reads to the primary assembly using Minimap2 v.2.28 (Li 2018) and sorted the alignment by coordinates with SAMtools v.1.9 (Danecek *et al.* 2021). We passed the resulting sorted bam file and the MegaBLAST output into the tool BlobTools v.1.0 (Laetsch and Blaxter 2017) to examine the contigs for taxonomic identification, coverage, and guanine and cytosine (GC) content. In particular, we were interested in contigs labeled as members of the phylum “Pseudomonadota,” showing higher coverage than the core genome, and distinct GC content. We then took the genomic sequences of these contigs and manually BLASTed them against the NCBI database to identify the specific bacteria species. Putative bacterial contigs were recorded as those that hit as bacteria via BlobTools annotation, those that were assembled as circular DNA (denoted with a “c” at the end of the contig number), or both (Table 1, columns 1 and 2). Since some of these hits represent distinct bacterial genetic sequences, while others may be the result of horizontal gene transfer from bacteria to mealybug (Husnik *et al.* 2013), we did not remove any contigs at this time. After Hi-C scaffolding and manual curation steps previously listed, we repeated the BlobTools and manual BLAST process on the final curated scaffolded assembly. We tracked whether sequences flagged as bacterial were incorporated into the core chromosomal scaffolds (as expected of horizontally transferred genes) or remained as distinct sequences (as expected of true symbiont genomes). Finally, we used NCBI's contaminant screening tool during assembly upload as a final piece of evidence for endosymbiont sequences. We removed the latter prior to archiving the genome sequence for public use but present those symbiont sequences here as a Supplementary file.

**Table 1.** Identification of the endosymbiotic bacteria found within the genome assembly of *Nipaecoccus viridis*.^a^

Primary assembly contig	Scaffold	BLAST identification	Percent identity (%)	BLAST query coverage (%)	Length (bp)	GC content (%)	Coverage (x)	Final assignment
ptg000042c	Scaffold_8	*Sodalis praecaptivus*	97.84	87	1,859,166	54.3	53	Putative symbiont, removed from assembly
ptg000152c	Scaffold_7	*Sodalis glossinidius*	92.58	59	2,030,015	54.7	51	Putative symbiont, removed from assembly
ptg000259l	Scaffold_25	*Candidatus Tremblaya princeps*	5.64	100	130,203	58.0	291	Putative symbiont, removed from assembly
ptg000296l	Scaffold_6	*Proteus appendicitidis*	73.65	5	2,149,523	39.2	19	Putative symbiont, removed from assembly
ptg000316l	Scaffold_3	*Planococcus citri*	92.21	17	110,262	31.0	11	Likely horizontal gene transfer, retained
ptg000798l	Scaffold_2	*Planococcus citri*	88.68	2	86,036	31.5	11	Likely horizontal gene transfer, retained
ptg001088l	Scaffold_73	*Burkholderia pseudomallei*	89.61	0	53,456	49.3	11	Unclear assignment, retained
ptg001145l	Scaffold_135	*Candidatus Tremblaya princeps*	94.90	90	35,172	57.8	113	Putative symbiont, removed from assembly
ptg001360c	Scaffold_268	*Candidatus Tremblaya princeps*	95.74	99	20,985	57.3	204	Putative symbiont, removed from assembly
ptg001498l	Scaffold_162	*Sodalis glossinidius*	92.58	34	30,728	51.3	133	Putative symbiont, removed from assembly
ptg001522l	Scaffold_270	*Candidatus Sodalis pierantonius*	93.59	31	20,905	49.8	114	Putative symbiont, removed from assembly
ptg001825l	Scaffold_83	*Arsenophonus nasoniae*	74.75	6	47,879	35.4	30	Unclear assignment, retained
ptg001995l	Scaffold_464	*Sodalis glossinidius*	93.05	66	14,180	54.7	26	Putative symbiont, removed from assembly

^a^Additional information includes the contig location within the primary assembly, scaffold location of the contig, and length of the bacterial segment. Contig names end with either “c” for circular or “l” for linear based on primary assembly.

### Gene annotation

Due to the need for a large number of individuals for the genome assembly, we lacked the tissue samples necessary to generate RNAseq datasets, so we used a de novo gene annotation approach with Helixer (Holst *et al.* 2023). This is a novel machine-learning tool that only requires a genome sequence and a general lineage as input; repeat masking and RNA evidence are not required. For our run, we used “invertebrate” as the lineage, specifically the invertebrate_v0.3_a_06000.h5 model. This tool and specific lineage have previously been used to annotate another scale insect with success (Mongue *et al.* 2024a). To assess this annotation, we ran BUSCO to identify orthologous proteins in our annotation using the hemiptera_odb10 dataset.

### Repeat masking

For the characterization of repeats, we used a combination of tools. We first used RepeatModeler v.2.0 (Flynn *et al.* 2020) to model de novo repeats, including long terminal repeats with the “-LTRStruct” parameter. This provided us with a set of repeats unique to *N. viridis*; these repeats were then added to a larger custom library. This custom library included repeats from the 2020 Repbase arthropod and hemipteran repeat databases (Bao *et al.* 2015), and other repeats recorded in the genomes of different scale insects, also identified through Repeatmodeler—namely, the cottony cushion scale *Icerya purchasi* (Maskell) (Mongue *et al.* 2024b), citrus mealybug *Planococcus citri* (Risso 1813) (Ross *et al.* 2024a), the Chinese wax scale *Ericerus pela* (Chavannes) (Yang *et al.* 2019), and the tuliptree scale *Toumeyella liriodendri* (Gmelin) (Mongue *et al.* 2024a). In this way, we created a library of repeats from both our target species and related insects. This custom database was imported into RepeatMasker v.4.0.9 (Smit *et al.* 2019) for the generation of a soft-masked assembly and the summary of our repetitive elements identified in *N. viridis.*

## Results and discussion

### Initial genome size estimates

For our PacBio HiFi long reads, we received 2 runs of the same genomic library sequenced on 2 separate single molecule, real-time cells, the first yielding 4.5 Gb of read data and the second run yielding 2.2 Gb of read data for a combined total of 6.7 Gb (raw data accessions found in Table 2). We found 1 peak in our k-mer coverage curve at a sequencing depth of ∼10× and estimated the genome to be 340 Mb. There was a steep decline until reaching a coverage depth of 4×, which represented the erroneous k-mers, those that contain errors from sequencing.

**Table 2.** Location of assembly data for *N. viridis.*

Data	Accession (Bioproject: PRJNA1104600)
PacBio HiFi reads	SRR28800798
Illumina Hi-C reads	SRR28800797
Assembly	GCA_052327235.1

### Sequencing and assembly

Chromosome number is consistent within Pseudococcidae, typically n = 5 (as reviewed in Gavrilov-Zimin *et al.* 2015) including *N. viridis* (Parida and Moharana 1982; Nur *et al.* 1987). As expected, based on this cytogenetic work, we assembled 5 haploid chromosomes. Following our final curation, we placed 91.2% of the assembly to our 5 chromosomal scaffolds. In our final curation steps, we made 4 manual adjustments to our assembly using the Juicebox v.2.3.0. tool (Robinson *et al.* 2018). These adjustments were made to realign contigs to their correct chromosomal placements based on the frequency of interactions between different regions in the assembly. After the manual curation, we found our final assembly yielded a BUSCO score of 89.9% completeness (85.4% single copy, 4.5% duplicated), 2.4% fragmented, and 7.7% missing (Table 3).

**Table 3.** Assembly statistics of our primary HiFi assembly, Hi-C scaffolded assembly, final curated assembly, and the final decontaminated assembly.^a^

Assembly statistics	Primary HiFi assembly (Hifiasm)	Hi-C scaffolded assembly (YaHS)	Final curated assembly	Final decontaminated assembly
Total length (bp)	289,586,077	289,742,477	289,742,677	283,451,800
Sequence count	2,067	552	556	547
N50	257,618	53,098,826	53,098,826	53,098,826
L50	340	2	2	2
GC content (%)	31.57	31.57	31.57	31.17
BUSCO completeness score (hemipteraodb10, n = 2510)	C: 89.9% (S: 85.1%, D: 4.8%), F: 2.5%, M: 7.6%, n: 2,510 2,255 complete BUSCO (C) 2,135 complete and single-copy BUSCOs (S) 120 complete and duplicated BUSCOs (D) 62 fragmented BUSCOs (F) 193 missing BUSCOs (M) 2,510 Total BUSCO groups searched	C: 89.9% (S: 85.4%, D: 4.5%],): 2.4%, M: 7.7%, n: 2,510 2,256 complete BUSCOs (C) 2,143 complete and single-copy BUSCOs (S) 113 complete and duplicated BUSCOs (D) 61 fragmented BUSCOs (F) 193 missing BUSCOs (M) 2,510 total BUSCO groups searched	C: 89.9% (S: 85.4%, D: 4.5%), F: 2.4%, M: 7.7%, n: 2,510 2,256 complete BUSCOs (C) 2,143 complete and single-copy BUSCOs (S) 113 complete and duplicated BUSCOs (D) 61 fragmented BUSCOs (F) 193 missing BUSCOs (M) 2,510 total BUSCO groups searched	C: 89.8% (S: 85.6%, D: 4.2%), F: 2.4%, M: 7.8% , n: 2,510 2,255 Complete BUSCOs (C) 2,149 complete and single-copy BUSCOs (S) 106 complete and duplicated BUSCOs (D) 61 fragmented BUSCOs (F) 194 missing BUSCOs (M) 2,510 total BUSCO groups searched

^a^Details include assembly size, contiguity, and BUSCO completeness.

Despite an initial genome estimated size of 340 Mb, our assembled genome size was smaller at 289.7 Mb. The discrepancy in sizes is attributable to the naive way in which our k-mer counting assesses genome size. The calculations we performed simply estimate the total length of unique sequence in the raw data, regardless of source; confounded in the k-mer based estimate are the endosymbiont genomes, any heterozygous regions across our pooled sample, or potential error-reads given the relatively low coverage.

While some scale insects can have massive genomes, such as the cottony cushion scale (*I. purchasi*) with a genome over 1,000 Mb (Mongue *et al.* 2024b), mealybug species typically range from 300 to 400 Mb. This can be seen in the citrus mealybug, *P. citri*, which has a genome size of 403.6 Mb (Ross *et al.* 2024a), and the New Zealand flax mealybug, *Balanococcus diminutus* (Leonardi), which has a size of 313.1 Mb (Ross *et al.* 2024b), and fall in the larger end of this range. Other species like the cotton mealybug, *Phenacoccus solenopsis* (Tinsley, 1898), have a more comparable size at 292.5 Mb (Li *et al.* 2020) to that of *N. viridis*, putting our genome size for *N. viridis* to be in the lower range of the recorded genome sizes for a mealybug species.

### Endosymbionts

#### BlobPlot results

**Fig. 3.**
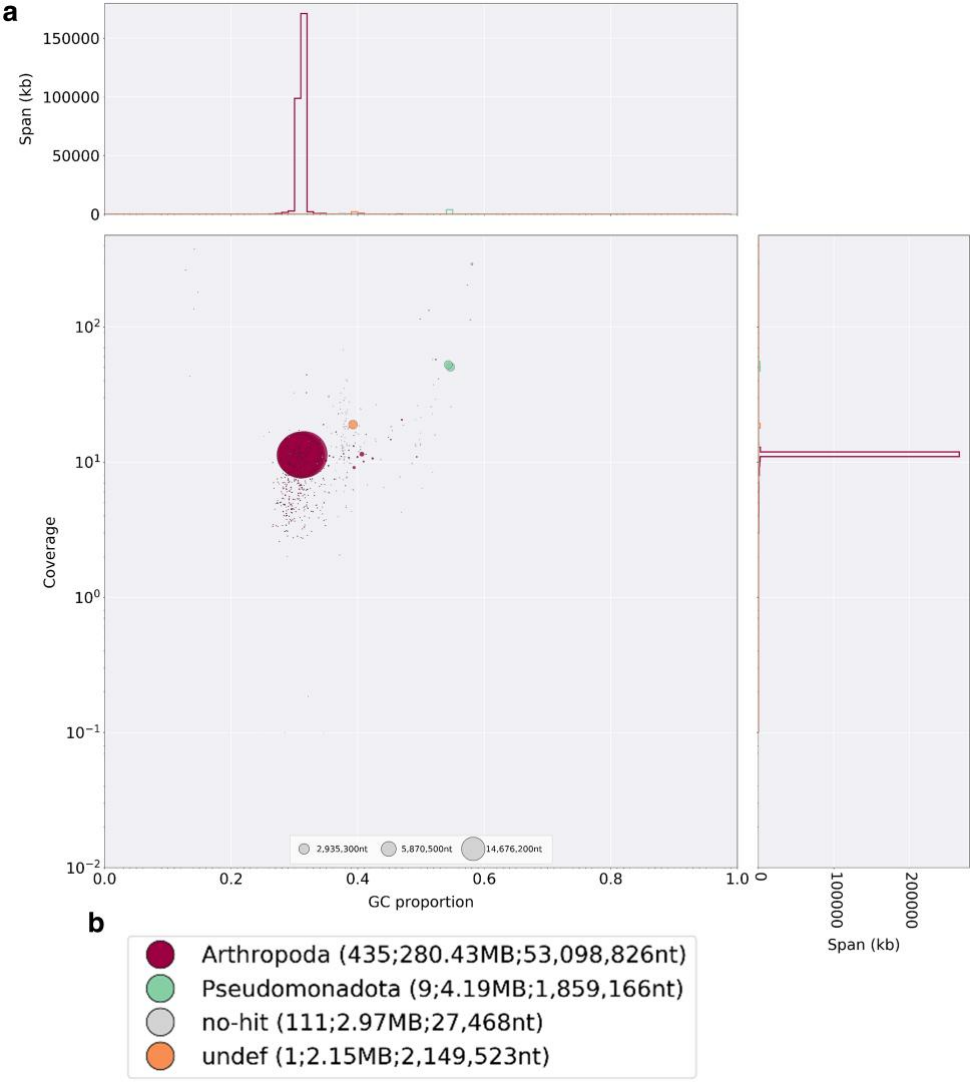
a) Blobplot visualization of the final curated assembly of *N. viridis*. Scaffolds are identified by the classification level of “Phylum” by color, identified in the legend (b) and further can be examined for their differences in coverage (y-axis) and GC content (x-axis). Blob sizes are proportional to scaffold length, with the sizes reflected by the size of the circle in the scale bar on the x-axis, (2,935,300, 5,870,500, and 14,676,200 nt). Endosymbiont sequences are expected to differ from the core mealybug genome in coverage, GC content and taxonomic identification.

In the BlobPlot visualization of our final curated assembly, we identified 5 chromosomal scaffolds as seen in the large, overlapping dark maroon color classified as “Arthropoda” (Fig. 3). We also saw scaffolds of potential bacterial sequences, colored in teal, and labeled as “Pseudomonadota.” These 2 groups differ not only in coverage but the GC proportion as well, with the bacterial segments having a higher coverage and a higher GC proportion, around 0.55 compared to the chromosomal scaffolds at a proportion around 0.31. Higher coverage suggests that more of these molecules were sequenced than for the core mealybug genome. Some variation in sequence coverage is random and natural to the sequencing process, but the higher pattern suggests that these sequences were in higher copy number than the core genome, as expected of the numerous bacterial cells that exist within each mealybug cell in their symbiotic tissue, the bacteriome organ (Husnik and McCutcheon 2016). Thus, these differences are suggestive but not conclusive of endosymbiont or other symbiont sequencing. To identify the endosymbionts, we further explored sequences as follows.

#### Identification of endosymbionts

In the primary genome assembly, we identified a total of 13 potential bacterial sequences, with 3 being assembled into circular contigs (Table 1, column 1), as expected of most bacterial chromosomes. In manual BLAST searching of NCBI's database, one of the circular contigs hit to *Tremblaya,* and the other 2 hit to different *Sodalis* species. Specifically, *Tremblaya princeps* is a known endosymbiont of other members of Pseudococcidae, including *P. citri* and *Pseudococcus longispinus* (Husnik and McCutcheon 2016). In tracking these contigs into our scaffolded assembly, all 3 sequences were recovered in the curated assembly as separate scaffolds (i.e. not assembled with other sequences). With this evidence taken together, these scaffolds represent the highest-confidence endosymbiont sequences.

An additional 5 contigs had BLAST hits to symbionts, did not assemble further with Hi-C data, but were initially assembled as linear sequences. Although we expect circular bacterial assemblies, the weight of evidence suggests that these are (partial) bacterial sequences as well. One contig, “ptg000296l” despite having a lower query coverage had an above average percent identity (73.65%), that hit to *Proteus appendicitidis* by NCBI BLAST. When uploading our genome to the NCBI database, their filtering program recognized this sequence as a bacterial strain to remove from the assembly as well. We excluded these 9 scaffolds from the core genome for archiving purposes as they are almost certainly not *Nipaecoccus viridis* sequences. We include them as a Supplementary fasta file for those interested but do not consider the endosymbionts in greater detail here. Of the remaining 4 potential bacterial contigs, we found 2 that assembled into the chromosomal scaffolds (scaffolds 1 to 5). The contig “ptg000798l” was placed on scaffold_2 and “ptg000316l” was found in scaffold_3 based on the Hi-C contact with other core genomic sequences. When BLASTing these sequences against NCBI's database, the top hits were not bacterial species, but rather another mealybug species, *P. citri*. The similarity to this mealybug and contact with *N. viridis* sequences suggest that these sequences are part of the core mealybug genome. Thus, the similarity to bacteria in the BlobPlot could represent horizontal transfer of genes from bacteria to insect, which has been reported in other mealybugs (Husnik and McCutcheon 2016). As such, we left these sequences intact as part of the mealybug genome.

Finally, 2 putative contigs, “ptg001088l” and “ptg001825l,” were found to be identified as the bacterial species *Burkholderia pseudomallei* and *Arsenophonus nasoniae*, respectively. However, in the manual BLAST step, both contig sequences were found to have very low (<10%) query coverages in the identification of these species and both had passed NCBI's contaminant screening on genome upload, so we did not remove these sequences from our core genome assembly.

### Gene annotation

We identified a total of 15,370 protein-coding genes within our genome for *N. viridis* using the Helixer tool (Holst *et al.* 2023). This annotation showed similar yet marginally better completeness to the BUSCO search of the genome assembly itself. Overall, we obtained 90.6% completeness (86.3% single copy, 4.3% duplicated), 1.5% fragmented, and 7.9% missing. By this metric, Helixer appears to have performed at least as well as more traditional evidence-based annotation methods used in other scale insects. By comparing *N. viridis* to other members of Pseudococcidae, we find that our recorded number of genes is on the lower end. Using the web resource Mealybug Base (https://ensembl.mealybug.org/index.html), we note that *P. citri* was recorded to have 40,620 genes and the longtailed mealybug, *P. longispinus* (Targioni Tozzetti), was recorded to have 20,766 genes. Both of these species utilized Ensembl Genebuild (Dyer *et al.* 2025) but on much more fragmented genomes. Another scale insect, the tuliptree scale (*T. liriodendri*), which was also annotated using Helixer, was found to have 16,580 protein-coding genes (Mongue *et al.* 2024a), which is comparable to the number found in *N. viridis*. Further RNA evidence will allow us to confirm our gene predictions for *N. viridis*.

### Repeat masking

We found that repetitive elements accounted for 91,843,934 bases or 32.40% of the sequence. A more in-depth breakdown of repeat content can be found in Table 4. In comparison to other species that have been sequenced to a similar level, *N. viridis* has a comparable repeat content to the cotton mealybug (*P. solenopsis*) which has a ∼290 Mb genome and ∼38% repetitive sequence (Li *et al.* 2020). In contrast, the larger soft scale *T. liriodendri* has a roughly 530 Mb genome with 64.69% of the sequence being repetitive (Mongue *et al.* 2024a), nearly double that of *N. viridis*, and consistent with a pattern of larger genomes having more repetitive elements (Yuan *et al.* 2024).

**Table 4.** Summary of the masked repeats in the *N. viridis* genome.

Repetitive element	Count	Sequence length (bp)	Percentage of sequence
Retro elements	19,367	8,797,510	3.10
SINEs	11	729	0.00
LINEs	8,659	3,578,092	1.26
L2/CR1/Rex	194	74,187	0.03
R1/LOA/Jockey	6,426	3,007,738	1.06
R2/R4/NeSL	106	13,176	0.00
RTE/Bov-B	407	139,248	0.05
L1/CIN4	3	210	0.00
LTR elements	10,697	5,218,689	1.84
BEL/Pao	1,080	553,216	0.20
Ty1/Copia	2,605	1,592,414	0.56
Gypsy/DIRS1	2,526	2,053,227	0.72
Retroviral	162	18,065	0.01
DNA transposons	13,454	3,709,106	1.31
hobo-Activator	1,560	311,013	0.11
Tc1-IS630-Pogo	2,104	472,297	0.17
MULE-MuDR	465	112,103	0.04
PiggyBac	561	171,750	0.06
Tourist/Harbinger	168	56,109	0.02
Other (Mirage, P-element, Transib)	95	48,375	0.02
Rolling-circles	8,065	1,475,777	0.52
Unclassified	314,860	79,337,318	27.99
Total interspersed repeats		91,843,934	32.40
Satellites	17	1,159	0.00
Simple repeats	136,474	4,512,679	1.59
Low complexity	29,952	1,423,408	0.50

## Conclusions

We report a chromosome-level assembly for *N. viridis*, an important widespread invasive agricultural pest. This assembly provides the necessary resources for further applied agricultural research and our knowledge about this species' path of invasion within Florida. Our final dataset provides a means of comparison to other worldwide records of this pest. Furthermore, this genome provides the baseline steps to other key features of this insect such as a deeper analysis of their unique sex determination system known as PGE, and a closer look at the unique composition of endosymbiotic bacteria housed within this insect.

## Supplementary Material

jkaf154_Supplementary_Data

## Data Availability

Data are publicly available through the National Library of Medicine: National Center for Biotechnology Information (NCBI) and the accession numbers for the genome assembly and raw sequence data can be found in Table 2. The annotation is hosted at https://github.com/TLiesenfelt/NiviGenome, and potential symbiont and contaminant sequences are provided as a Supplementary file. Supplemental material available at G3 online.
